# Tactile information counteracts the attenuation of rubber hand illusion attributable to increased visuo-proprioceptive divergence

**DOI:** 10.1371/journal.pone.0244594

**Published:** 2020-12-30

**Authors:** Piotr Litwin, Beata Zybura, Paweł Motyka

**Affiliations:** 1 Faculty of Psychology, University of Warsaw, Warsaw, Poland; 2 Polish Academy of Sciences, Institute of Philosophy and Sociology, Warsaw, Poland; Universitat de les Illes Balears, SPAIN

## Abstract

Sense of body ownership is an immediate and distinct experience of one’s body as belonging to oneself. While it is well-recognized that ownership feelings emerge from the integration of visual and somatosensory signals, the principles upon which they are integrated are still intensely debated. Here, we used the rubber hand illusion (RHI) to examine how the interplay of visual, tactile, and proprioceptive signals is governed depending on their spatiotemporal properties. For this purpose, the RHI was elicited in different conditions varying with respect to the extent of visuo-proprioceptive divergence (i.e., the distance between the real and fake hands) and differing in terms of the availability and spatiotemporal complexity of tactile stimulation (none, simple, or complex). We expected that the attenuating effect of distance on illusion strength will be more pronounced in the absence of touch (when proprioception gains relatively higher importance) and absent in the presence of complex tactile signals. Additionally, we hypothesized that participants with greater proprioceptive acuity—assessed using an elbow joint position discrimination task—will be less susceptible to the illusion, but only under the conditions of limited tactile stimulation. In line with our prediction, RHI was attenuated at the farthest distance only when tactile information was absent or simplified, but the attenuation was effectively prevented by the use of complex tactile stimulation—in this case, RHI was comparably vivid at both distances. However, passive proprioceptive acuity was not related to RHI strength in either of the conditions. The results indicate that complex-structured tactile signals can override the influence of proprioceptive signals in body attribution processes. These findings extend our understanding of body ownership by showing that it is primarily determined by informative cues from the most relevant sensory domains, rather than mere accumulation of multisensory evidence.

## Introduction

Sense of body ownership is no longer considered an invariant of human experience [1]. One fruitful paradigm that investigates its flexibility is the rubber hand illusion (RHI) [2], in which an artificial hand comes to be experienced as the participant’s own after a period of simultaneous brush stroking of the participant's hidden real hand and a visible dummy hand. The spatiotemporal coherence of stimulation appears to be a crucial condition for divergent visual, tactile, and proprioceptive signals to be integrated, and, consequently, for a sense of ownership over a fake limb to arise [3].

The exact way in which multisensory integration processes lead to the emergence of the rubber hand illusion remains a subject of heated debate (for a discussion see [4, 5]). One influential family of models are Bayesian causal inference models, which propose that the presence of the illusion depends on whether the brain infers that the visual and somatosensory signals stem from a common cause (for neurophysiological evidence of causal inference processes in non-human primates’ premotor cortex see [6]). Associations between sensory signals previously learned to frequently co-occur establish the prior probability of their shared origin, while likelihood is determined by the spatiotemporal congruence of visual, tactile, and proprioceptive signals. When the signals converge in space and time, the increased likelihood of observing such signals (given the assumption that they have a common cause) boosts the posterior probability of their common cause and thus supports the inference (and, as a result, the experience) of ownership over a fake hand. In a recent computational account [7], visuo-tactile synchrony and distance between the real and rubber hands are two major factors determining the inference of the commonality of signals. The distance between the hands accentuates the role of proprioception in the rubber hand illusion: greater discrepancies between visual and proprioceptive cues about the hand’s location weaken or break the RHI, particularly when the proprioceptive signals are of high precision. In other words, “the illusion is stronger the nearer the fake and real hand are to each other, [and] the noisier the proprioception modality is” [7, p. 19].

In our previous study [8], we aimed to experimentally examine these predictions. To manipulate the distance, we subliminally moved the participant’s hand at a very slow pace from the starting position (16cm between the real and rubber hands) to positions either closer (8cm) or farther away from (24cm) the rubber hand. RHI turned out to be comparably vivid at both distances, regardless of the participant’s individual proprioceptive precision (which was operationalized as the inverse variance of errors in an active arm reproduction task [9]). For a subjective measure of RHI strength, Bayes factor analyses provided substantial evidence for the null hypotheses, both when fitting unifactorial and full regression models. These results throw into question the relevance of 1) the degree of convergence of visual and proprioceptive spatial estimates and 2) proprioceptive precision for RHI induced visuo-haptically at distances shorter than 30cm (for reports of attenuation of RHI at larger distances see [10, 11]).

According to the extended Bayesian model of the RHI [5], the relevance of proprioception-laden factors may be diminished in the presence of touch. In the process of causal inference, cognitive systems use various sources of information, depending on their availability, reliability, and relevance for the task at hand. As for relevance, touch directly demarcates one’s body boundaries, as its receptor surface (skin space) is “physically co-extensive with the body itself” [12, p. 12]. Additionally, given that the skin space constitutes a high-resolution spatial map, the presence of spatiotemporally congruent visuo-tactile stimulation provides highly reliable sensory evidence for the hypothesis that the rubber hand is actually one’s own. Therefore, even if there is a noticeable discrepancy between visual and proprioceptive spatial estimates, visuo-tactile coherence renders it insignificant due to the superior precision of signals originating on the skin space. However, in the absence of tactile stimulation—when the RHI is elicited through the mere observation of a dummy—the inference should rely on the degree of visuo-proprioceptive convergence and the precision of unimodal visual and proprioceptive estimates (in line with the causal inference model of the RHI as proposed in [7]). On this basis, it could be hypothesized that, in the presence of touch, the convergence of visuo-proprioceptive estimates is still a ‘working’ factor, but due to its minimal influence on the final inference (favoring the separate causes hypothesis), it becomes virtually non-determinative of illusion strength. Visuo-proprioceptive convergence ceases to be a causal factor only in the case of complete absence of the proprioceptive input. Then, body attribution processes may be driven solely by visual body information, as shown by the studies carried out among patients suffering from spinal cord injuries at the cervical level [13, 14].

Furthermore, this compensatory effect of tactile information should depend on its complexity. Decreased complexity of spatiotemporal patterns of brush stroking—for instance, repetitive single-finger taps—renders visuo-tactile co-variance over space and time less informative (as illustrated by the fact that irregular and unpredictable stimulation patterns give rise to a more vivid illusion) [15]. Thus, we hypothesize that the magnitude of the effect of spatial distance on RHI strength will be proportional to the relative influence of proprioception, which should be greater when tactile information is more limited. Also, we expect to observe that the effect of spatial distance will be more pronounced for individuals with better proprioceptive abilities, but only when tactile information is absent or simplified. In the case of complex tactile stimulation, which is supposed to render the impact of visuo-proprioceptive estimates negligible, proprioceptive precision has been shown not to be predictive of RHI strength [8].

Finally, the relation between subjective reports of illusion strength and one of its classic behavioral proxies—proprioceptive drift [2]—should also depend on the availability and complexity of tactile information. Once the illusion has been induced, proprioceptive estimations of the position of one’s own hand are shifted towards the rubber hand and the magnitude of this shift tends to correlate with subjective RHI strength [10, 16]; however, the absence of such correlations is also frequently reported [17–21]. On this basis, it was argued that correlations are found only with the use of a particular measurement technique, that is, with the contralateral hand pointing at the position directly over the ipsilateral hand [22], or that proprioceptive drift may be a behavioral proxy of RHI only under specific circumstances, namely in experimental designs in which the participant’s hand is not displaced [23]. Alternatively, it might reasonably be argued that questionnaire ratings and proprioceptive drift capture different aspects of the RHI phenomenon [20]. From the perspective of probabilistic multisensory integration, determination of ownership (‘what are the parts of my body’) and location (‘where are the parts of my body’) are two different tasks for a cognitive system. While touch is relevant for the former, the latter is based only on visuo-proprioceptive estimates. Therefore, we hypothesize that correlations between subjective body ownership and drift should be particularly pronounced in the ‘no touch’ mode of RHI elicitation (i.e., when one is simply looking at a rubber dummy without receiving tactile stimulation) [7], because in this case the cognitive system uses the same visuo-proprioceptive estimates for both tasks.

In order to examine the previously-discussed predictions of the extended Bayesian model of the RHI [5], the subliminal displacement procedure [8, 23] was used to manipulate the distance between the participant’s hands (8 and 24 cm) and the RHI was elicited under conditions differing in terms of the availability and complexity of tactile stimulation (none, simple, complex). Additionally, we measured individual proprioceptive acuity. To ensure that the potential lack of effect of proprioception (cf. [8]) would be due to its irrelevance rather than an ill-chosen task, we decided to assess passive proprioception, as it is the most relevant in the conditions under which the RHI is generally assessed. The activation of several functionally distinct proprioceptive sensors, such as muscle spindles (major sensors of limb position and movement) or joint and skin mechanoreceptors, may differ depending on the kind of proprioceptive task used [24]. For example, in passive proprioceptive tasks, sensory feedback from cutaneous receptors is enhanced at the cost of input from muscle spindles. Therefore, active joint position reproduction (JPR) tasks may measure different aspects of proprioception than passive methods [25]; indeed, performances in threshold position/movement detection tasks and active reproduction tasks do not correlate [26], which suggests that ‘passive’ and ‘active’ proprioception subsystems should be distinguished. Therefore, while we used an active reproduction task (including arm flexions and abductions at the glenohumeral joint) in our previous study [8], in the present study we decided to use one which measures the elbow joint’s position sense threshold. In this task, the participant’s initial arm position is matched with the position of the arm during RHI elicitation and the experimenter moves the participant’s arm passively to the testing positions. We thereby ensured that the task assessing proprioceptive skills (precision) is maximally relevant to the sensory conditions accompanying RHI.

To sum up, we aimed to test three hypotheses concerning the interactions between visual, tactile, and proprioceptive cues in the shaping of body ownership. First, we expected that the attenuating effect of distance on RHI will be absent in the presence of complex tactile stimulation, diminished under the condition of limited tactile information, and most pronounced in the absence of touch. Second, we hypothesized that proprioceptive precision will be inversely related to RHI strength in the *no touch* and *simple touch* conditions—specifically, at the *far* distance between hands—whereas it will not have predictive value for the *complex touch* condition (at both distances) [8]. Third, we expected that the magnitude of correlation between proprioceptive drift and RHI strength will be inversely proportional to the amount of tactile information available.

## Materials and methods

### Participants

The sample comprised 58 participants (32 females, 48 right-handed; mean age 21.98, *SD* = 4.81, range: 19–30 years), a number slightly increased as compared to other studies concerning the role of distance in RHI (e.g., *N* = 40 [10]; *N* = 18 [27]; *N* = 55 [11]; *N* = 50 [8]). It is worth noting that previous studies mainly used (1) distances larger than 30 cm [10, 11, 27] (which may invite confounding factors responsible for the attenuation of RHI, such as placement of the rubber hand outside of peripersonal space or in the implausible position), (2) varied alignment axes (e.g., vertical [10] or distal [11]), and (3) unpredictable (complex) tactile stimulation patterns [8, 11, 27]. Due to these significant methodological differences, a proper power analysis based on expected (standardised) effect size could not be carried out prior to the experiment (particularly for *no touch* and *simple touch* conditions).

Participants were recruited via social media. We only recruited participants who claimed not to have a history of psychiatric or neurological conditions (due to diverse susceptibility to the RHI in populations with such disorders) [28]. All participants gave written informed consent before taking part in the study and received monetary compensation (40 PLN = ~ 9 ‎€). The study took 50 minutes to complete. The procedure was approved by the Ethics committee of the Faculty of Psychology of the University of Warsaw.

### Setup and tasks

#### Proprioceptive acuity assessment

**Fig 1:**
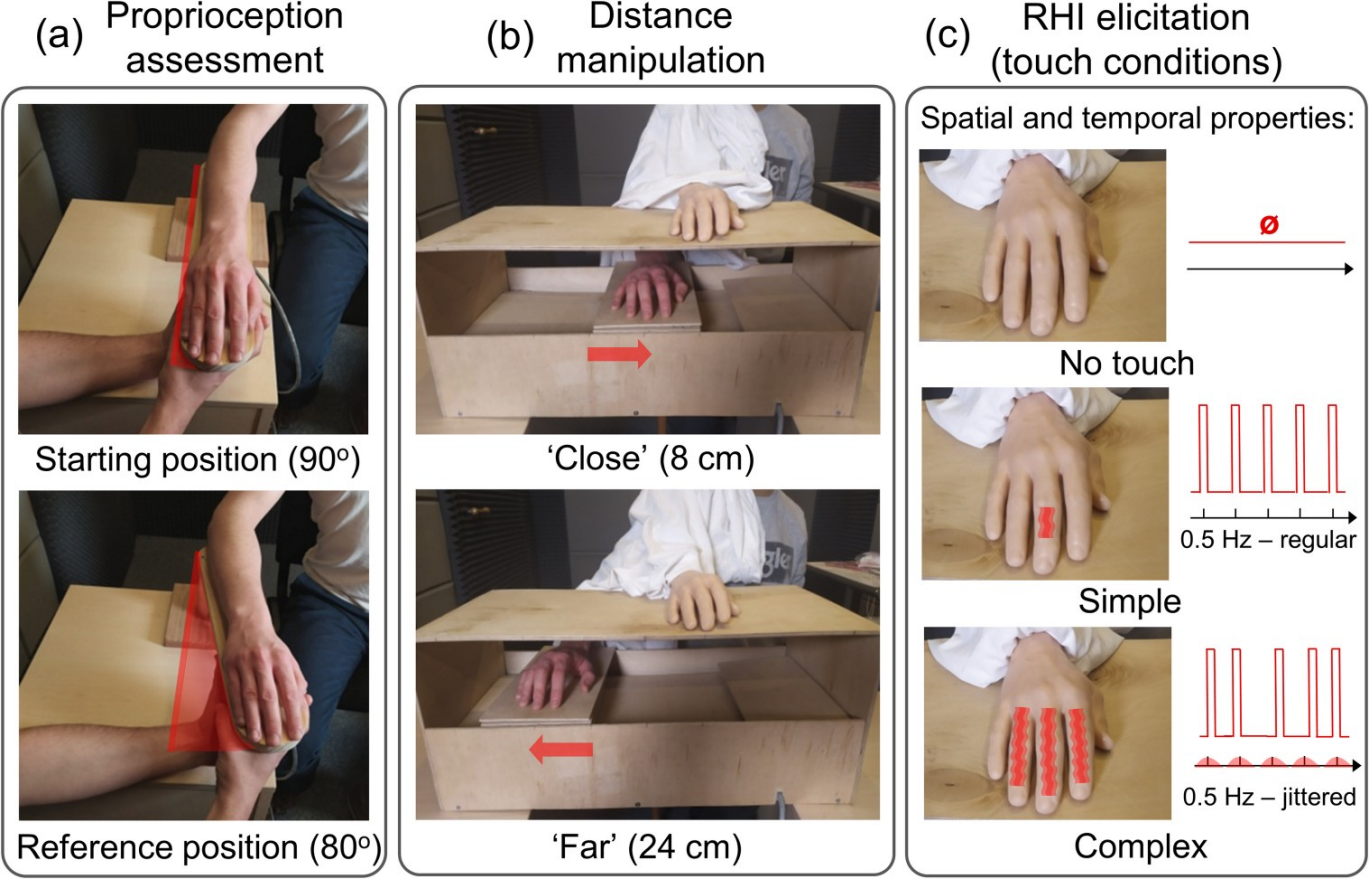
Experimental setup. (A) Proprioception assessment. The experimenter moved the armrest from the starting position (90^o^) to the two following positions: 1) the reference position (80^o^) and 2) the comparison position (ranging from 80^o^ to 86^o^ in 0.1^o^ steps). The participant’s task was to indicate which one of the two positions was closer to the body. (B) Distance manipulation. Before the illusion elicitation, the participant's real hand was displaced at a very slow pace (under the detection threshold) to position either close to or far from the rubber hand. (C) Elicitation conditions. In the no touch condition, the illusion was elicited through the sole observation of the rubber hand. In the two remaining conditions, RHI was induced with tactile stimulation. The brushstrokes varied with respect to their spatiotemporal complexity–were either repetitive and spatially limited (simple touch condition) or temporally jittered, unpredictable and spatially extended (complex touch).

Proprioceptive acuity was measured with a modified version of the psychophysical threshold method [26], which assesses the ability to differentiate between arm positions on a horizontal axis (angles of elbow joint flexion). Participants were blindfolded, sat in an upright position, and asked to place their right arm on a movable armrest. During each trial, the arm was initially placed in the starting position (elbow flexion = 90^o^; Fig 1A–upper panel). The starting position of the arm during the task was matched with the position of the arm during RHI elicitation. The trained experimenter moved the armrest toward the midline of the participant’s body in a circular motion (allowed by the manipulandum) to the two following positions in a counterbalanced order: 1) the reference position (elbow flexion = 80^o^; Fig 1A–bottom panel) and 2) the comparison position (ranging from 80^o^ to 86^o^ in 0.1^o^ steps). Each position was denominated by the experimenter as either the ‘first’ or the ‘second’. Then, the participant provided a verbal response (in a two-alternative forced choice task) indicating which of the two positions was closer to the body. No feedback was given to the participant. To ensure that the participants understood the task, two trial runs with easily noticeable differences between positions were conducted prior to the assessment.

Based on the correctness of the response, the psi-marginal adaptive staircase method [29] (as implemented in Python by [30]) determined the comparison position for each subsequent trial by increasing stimulus intensity (the difference between positions) after a wrong answer and decreasing it after a correct one. In this way, the adaptive algorithm converged towards the participant’s just-noticeable-difference (JND) threshold [31]. A uniform threshold prior and initial stimulus intensity (difference) set to 2.8^o^ were fed into the algorithm. To ensure that the participants maintained mental alertness throughout the task, breaks of one minute were taken after the 13^th^ and the 26^th^ trials (cf. [32]). After 40 trials, the algorithm outputs a logistic function fitted to the response data as well as just-noticeable-difference thresholds and estimates of the variability of the participant’s responses (represented as the slope of the logistic function) [31]. Proprioceptive accuracy was operationalized as being inversely related to just-noticeable-difference threshold and proprioceptive precision as being inversely related to the slope of the distribution. The assessment procedure lasted from 15 to 20 minutes.

#### Rubber hand illusion elicitation

For illusion elicitation, a realistic, life-sized, right male rubber hand was used (in our previous study, no differences in illusion strength were observed between genders when a male hand was used) [8]. Depending on the distance (D) condition, the participant’s real hand was subliminally moved from the starting position (16 cm away from the rubber hand) either to the *close* (8 cm away from the rubber hand; Fig 1B–upper panel) or *far* (24 cm away from the rubber hand; Fig 1B–bottom panel) position with the use of a displacement sheet. Then, the illusion was elicited in three touch-related (T) conditions: a no touch condition (visuo-proprioceptive, Fig 1C–upper panel) and two visuo-tactile conditions (consisting of the synchronous stimulation of the real and the rubber hands with a brush): simple touch (Fig 1C–middle panel) and complex touch (Fig 1C–bottom panel). The simple and complex conditions differed in the number of stimulated fingers (simple: one finger; complex: three fingers stimulated in a random sequence), temporal structure (simple: steady, regular stimulation with a frequency of 0.5 Hz; complex: irregular stimulation with beep onsets jittered by adding Gaussian temporal noise with a mean of 0 seconds and standard deviation of 0.2 seconds) [33], and spatial patterns of stimulation (simple: repetitive brushstrokes extending from the proximal to the middle phalanx of the finger; complex: brushstrokes of varying length extending from the proximal to the distal phalanges). The stimulation in all conditions lasted 90 seconds and was standardised by using auditory beeps, audible only to the experimenter, to signal the timings of the strokes. As in the previous study [8] we used the audio tracks developed by Fuchs et al. [33] with slightly extended beep durations.

#### Rubber hand illusion questionnaire

To measure the subjective strength of the illusion, a six-item questionnaire was used. The questionnaire was based on that of Longo et al. [34]. All questions began with the phrase “During the block I felt as if…” and answers were given on a seven-point Likert-type scale from -3 (*I didn’t feel like that at all*) to 3 (*I felt like that very strongly*). We had to exclude the conventional illusion items concerning tactile experiences (e.g., “…the touch I felt was caused by the paintbrush touching the rubber hand”)—even though their mean values are usually the highest (cf. [34])—as they would not relate to the no touch condition. Instead, to measure the illusion strength with the same items across all conditions, the three non-touch-related items with the highest loadings on the embodiment factor were selected. Control items were used in order to account for the potential tendency of participants to assent to the given statements. The selection criterion here was the lowest mean ratings of the rubber hand illusion strength in Longo et al.’s [34] study, given that the chosen illusion items do not tend to yield particularly high scores (average ratings tend to oscillate around 0). The selected items were translated into Polish and pre-tested during a pilot study (*N* = 10) to ensure that they yield comparably low mean ratings. The items were read aloud to the participant in a randomized order. All items are presented in Table 1.

**Table 1:** Illusion strength questionnaire items.

During the block I felt as if. . .	
Illusion items	Control items
Q1. . .the rubber hand belonged to me.	Q4. . .I had more than one right hand at a time.
Q2. . .the rubber hand was my hand.	Q5. . .the rubber hand was moving toward my hand.
Q3. . .the rubber hand was part of my body.	Q6. . .I had the sensation of pins and needles in my hand.

#### Proprioceptive drift assessment

For proprioceptive drift assessment, a modified version of the procedure from Holle et al. [35] was used, adapted to our rubber hand illusion elicitation setup. From a variety of proprioceptive drift measures (cf. [22]), a visual judgment task was identified as being the most suitable, given that our hypotheses dealt with visuo-proprioceptive integration. Using, for instance, a contralateral matching task would solely engage proprioception. In our task, the participant’s hand was placed on the bottom surface of the shelf and the rubber hand was hidden with a wooden cover. A ruler was placed at the farthest edge of the top surface. The participant’s task was to verbally indicate the number under which they *felt* (cf. [36]) the middle finger of their real hand was placed (with 0.5 cm accuracy). Proprioceptive drift was measured twice in each condition: before (pretest) and after (posttest) elicitation of the illusion. In order to avoid the participant having been primed by the pretest number, the ruler’s posttest position was randomly shifted relative to its pretest position. The position of the middle finger for both the close and far conditions was marked on the wood on the experimenter’s side of the shelf so as to compare the participant’s answer with the correct number without the experimenter’s gaze indicating the actual position of the hand. The illusion-induced proprioceptive drift value was calculated as the difference between the posttest and the pretest drifts.

### Procedure

Participants were sent an information document beforehand to inform them about the experimental procedure and exclusion criteria. After signing the informed consent sheet, the participant entered the soundproof laboratory in which the experiment was conducted. In the first part of the experiment, the proprioceptive acuity of the participant was assessed. Then, the rubber hand illusion was elicited in a within-subject 2 (D1: close *vs* D2: far) x 3 (T1: no tactile stimulation *vs* T2: simple tactile stimulation *vs* T3: complex tactile stimulation) design. The participant was seated in front of a two-level shelf, opposite the experimenter. The rubber hand was placed on the top surface, directly in front of the participant’s right shoulder, while their real hand was hidden beneath on a movable surface which allowed the experimenter to manipulate the distance between hands (Fig 1B). The space between the participant’s neck and the rubber hand was hidden with a textile material. A wooden cover was placed upright on the shelf to prevent participants from seeing the experimenter’s left hand stimulating their real hand. It was consequently used in the no-touch conditions so that the procedure was identical across all conditions. The real and the rubber hand were separated by 12.5 cm on the vertical axis and by 8 cm (close condition) or 24 cm (far condition) on the horizontal axis. As in our previous study [8], the real hand was subliminally moved from the starting position (16 cm away from the rubber hand) either to the close (8 cm) or far (24 cm) position with the use of a displacement sheet composed of two plywood sheets separated by vibration isolation elements and operated by hidden electrical engine. Positions were set and movement was initiated with the use of an external driver visible only to the experimenter. The 8 cm displacement took 90 seconds, which gives a proprioceptively undetectable velocity rate of 0.9mm/s [37]. Additionally, we aimed to minimize the possibility of alternative ways of movement detection. A vibration isolation system composed of rubber and foam elements supporting the upper surface of the displacement sheet precluded detection of vibration. Two noisy fans were running throughout the whole experiment so as to drown out the sound produced by the shelf being displaced. During the displacement, the textile material and wooden cover were arranged, instructions repeated, and demographic data collected.

The order of the conditions was counterbalanced between participants in the following manner: first, the order of distance (D) conditions (D1: far and D2: close) was randomly drawn, and then the sequence of tactile (T) conditions (T1: none; T2: simple; and T3: complex) was randomized within each of the distance conditions. After threefold RHI elicitation at the first distance condition, the displacement sheet was reset to the starting position. Then, the participant's hand was analogously displaced to the remaining (counterbalanced) distance condition. Thus, the participant’s hand was displaced twice during the experiment: once at the beginning and later at its midpoint. We decided to reduce the number of mechanical displacements (i.e., to not use a fully randomized order for all six conditions) to minimize the long breaks throughout the session and to reduce the participants’ potential suspicion about the purpose of the study. In order to avoid habituation or transfer effects impinging on the illusion, after each condition participants were encouraged to move their right hand (e.g., clench and unclench or slightly shake) while keeping it on the shelf. Before and after eliciting the illusion in each condition, the participant’s proprioceptive drift was measured. After each posttest proprioceptive drift measurement, a six-item illusion strength questionnaire was read aloud to them and their answers were registered. Finally, they were asked whether they had noticed the movement of the shelf (4 out of 58 had, 2 of them were excluded from further analyses as illusion non-responders, see below) and were then debriefed and compensated for participating. The whole procedure lasted 50 minutes.

### Data analysis

We adopted the most widely used method of quantification of subjective RHI strength [22] and computed illusion and control scores by averaging three ownership (Q1–Q3) and the control (Q4–Q6) questionnaire items, respectively. Only participants who obtained an averaged illusion score of ≥ 1 in at least one of the six conditions were classified as illusion responders (cf. [10, 11]) and entered the final analysis. A total of 12 out of 58 participants did not experience the RHI in any of the six conditions, which resulted in an exclusion rate of 20.6%, similar to those reported elsewhere in the literature (e.g., 28% [38]; 22.5% [10]; or 18.2% [39]). Given that non-normal distributions of RHI strength were observed for all six conditions (see S1 Fig), we employed commonly used non-parametric tests [10, 11, 27] to examine the effects of distance and touch availability on subjective RHI strength. Continuity-corrected Wilcoxon signed-rank tests were used for pairwise comparisons [11] and matched-pairs rank-biserial correlations [40] computed as effect sizes. Additionally, we performed Bayesian Wilcoxon signed-rank tests to determine the relative support for alternative and null hypotheses in each tactile condition. Given that we lacked proper justification for informed prior specification, we used the default Cauchy prior with width r set to 0.707 (1/√2) [41]. Bayes factor robustness was further validated with analyses using narrow (r = 0.354), wide (r = 1) and ultrawide (r = √2) priors. BF analyses were reported and interpreted in accordance with the recent guidelines [41]

As common non-parametric methods do not permit the conclusive examination of interaction effects, linear quantile mixed models (LQMM) were used in the complementary analysis [42]. This statistical approach to dependent data makes no assumptions regarding the distribution of the outcome variable. This is of paramount importance in the context of the RHI, since skewed illusion score distributions frequently violate normality (as indeed observed in our study). Additionally, quantile regression permits the estimation of effects across a range of the distribution, which allows more fine-grained discrimination of manipulation effects, that is, among subjects more or less prone to the RHI in general. In our analyses, linear quantile mixed models were fitted for three quartiles of RHI strength: among so-called “weak” (25^th^ percentile), “moderate” (50^th^ percentile), and “strong” (75^th^ percentile) illusion responders.

Linear quantile mixed models were fitted using the *lqmm* package [43] for R. The obtained coefficients represent *the expected change* in the value of the outcome variable at the *n*^th^ percentile of subjective RHI strength for each unit of change in the predictor (assuming, in interaction designs, the other predictor to be at the baseline level). For interactions, the coefficient reflects *the difference in the change* of RHI strength compared to the change when the interacting variable is at the baseline level [44]. We calculated 95% confidence intervals by bootstrap, with an increased number of iterations (200) to provide more stable estimates of parameters. Since the distribution of the outcome variable (illusion score) was discrete and bounded between -3 and 3 (in steps of 0.333…), the dependent variable was transformed to a logit scale [45] with the use of the following formula:

$$(1)\;\mathrm{Illusion}\;\mathrm{logit}=\log\left(\frac{\mathrm{illusion}\;\mathrm{score}+3.001}{3.001-\mathrm{illusion}\;\mathrm{score}}\right)$$

The small quantity (0.001) added to the maximum illusion score allowed this transformation to be computed for all values. For ease of interpretation, coefficients and confidence inteval bounds were transformed back to the original scale [46] as follows:

$$(2)\;\mathrm{Illusion}\;\mathrm{score}=3.001\times \frac{e^{\mathrm{illusion}\;\mathrm{logit}}-1}{e^{\mathrm{illusion}\;\mathrm{logit}}+1}$$

Following Filipetti and colleagues [47] we excluded drift measurements with a score below -2 or above 2 *SD* from the whole sample mean (including non-responders) as outliers. The same exclusion criterion was adopted for proprioceptive accuracy (JND threshold) and precision (slope of the distribution) measures [26]. In sum, 14 out of 276 drift measurements (from 10 participants and all different illusion elicitation conditions), 3 out of 46 threshold measurements (two outliers; data from one subject missing due to measurement device failure), and 5 out of 46 slope measurements (four outliers; data from one subject missing due to measurement device failure) did not enter the final analysis. All cases were excluded pairwise. Pearson correlations were run for normally distributed variables. In the case of dissimilar (e.g., skewed) distributions, non-parametric correlations were used.

All reported analyses are two-tailed and were performed in R 3.5.3. software. All preprocessed data and the R data analysis code are available on GitHub at https://github.com/Pawel-Motyka/RHI_TD.

## Results

The questionnaire items were found to have been appropriately chosen because: 1) the illusion scores were much greater than the control scores in all conditions (all *ps* < .0032) and 2) averaged control scores did not significantly differ across conditions, as shown by Friedman’s test: *χ*^*2*^(5, *N* = 46) = 3.51, *p* = .622 (descriptive statistics and bar plots are presented in S1 Table and S2 Fig).

### The influence of the interplay between distance and touch availability on RHI strength: Non-parametric analyses

**Fig 2:**
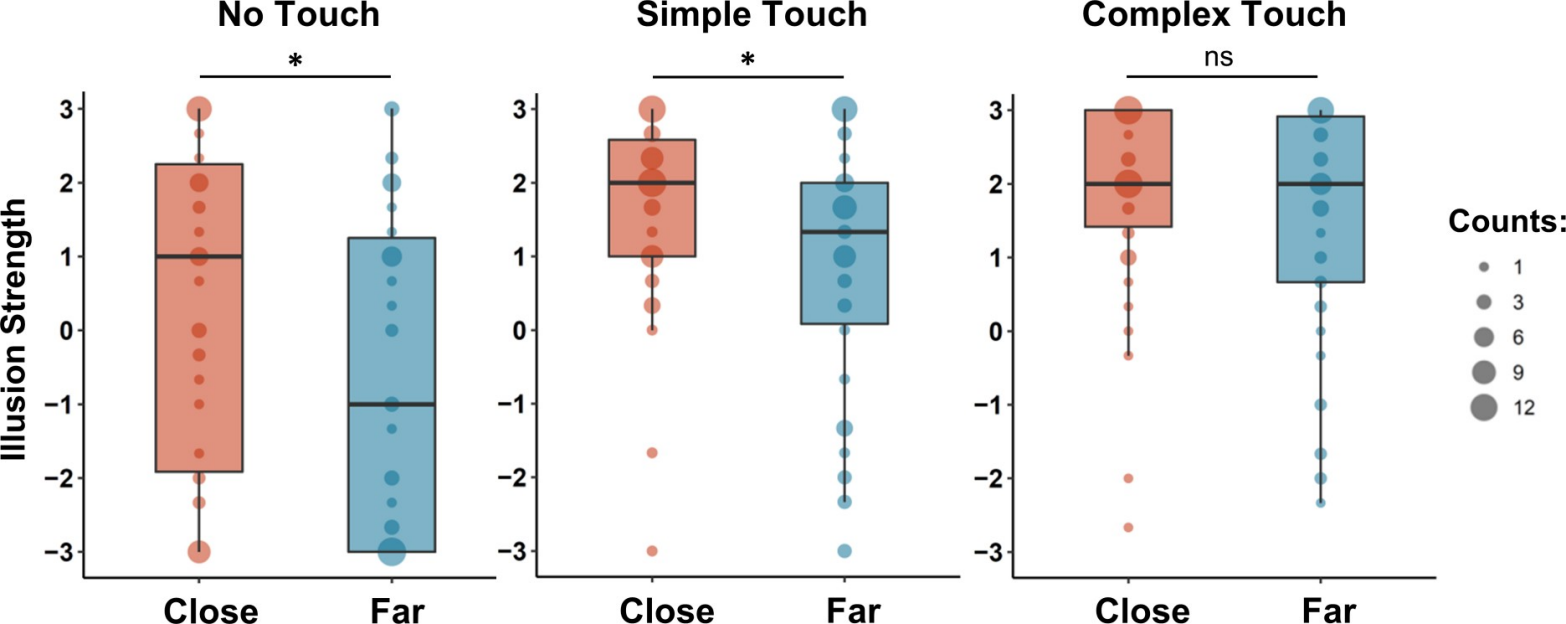
Changes in the effect of distance between hands on RHI strength due to the amount of tactile information present during RHI elicitation. The boxplots depict medians of subjective RHI strength (bold horizontal lines), upper values of the first and third quartiles (lower and upper sides of the box), and interquartile ranges for different elicitation conditions. The illusion was attenuated at the farther distance only in the conditions of absent or simplified tactile stimulation; no significant differences were observed for complex stimulation patterns. * *p* < 0.05.

Non-parametric analyses revealed a pattern of results close to the hypothesized interaction (Fig 2). When the RHI was elicited through mere observation (T1), the illusion was weaker at the larger distance between hands (D2: *Me* = -1) than at the smaller distance (D1: *Me* = 1): *Z* = -2.57, *p* = 0.010, *r* = 0.500, *BF*_*10*_ = 7.94, *δ* = 0.416 [95% CI: 0.123, 0.724]. Similarly, in the simple touch condition (T2), attenuation at the larger distance was observed relative to the shorter position: *Z* = -2.45, *p* = 0.014, *r* = 0.464, *BF*_*10*_ = 7.41, *δ* = 0.411 [95% CI: 0.119, 0.710] (D2: *Me* = 1.33; D1: *Me* = 2). However, in case of complex tactile stimulation (T3), there was no evidence for differences in RHI strength between the distance conditions: *Z* = -1.48, *p* = 0.138, *r* = 0.312, *BF*_*10*_ = 0.47, *δ* = 0.218 [95% CI: -0.064, 0.508] (D2: *Me* = 2; D1: *Me* = 2). Obtained Bayes factor values indicated moderate evidence for the alternative hypothesis in the case of no touch (*BF*_*10*_ = 7.94) and simple touch (*BF*_*10*_ = 7.41) conditions, and weak evidence for the null hypothesis in the case of complex touch condition (*BF*_*10*_ = 0.47). There remains some degree of uncertainty regarding effect sizes in the no touch and simple touch conditions, given the relatively wide extents of corresponding 95% credible intervals (weak-to-medium effect size).

Across a wide range of Cauchy prior widths, Bayes factors appeared to be relatively stable. In the no touch (T1) condition, we observed moderate evidence for the alternative hypothesis under all prior specifications–narrow prior: *BF*_*10*_ = 8.69, *δ* = 0.365 [95% CI: 0.075, 0.681], wide prior: *BF*_*10*_ = 7.15, *δ* = 0.437 [95% CI: 0.126, 0.725], ultrawide prior: *BF*_*10*_ = 7.00, *δ* = 0.450 [95% CI: 0.150, 0.764]. Similarly, moderate evidence for RHI attenuation at the larger distance was found in the simple touch (T2) condition–narrow prior: *BF*_*10*_ = 5.89, *δ* = 0.353 [95% CI: 0.055, 0.659], wide prior: *BF*_*10*_ = 7.07, *δ* = 0.425 [95% CI: 0.128, 0.728], ultrawide prior: *BF*_*10*_ = 4.64, *δ* = 0.428 [95% CI: 0.126, 0.732]. In the complex touch (T3) condition, we obtained inconclusive results when the narrow prior was used: *BF*_*10*_ = 0.82, *δ* = 0.178 [95% CI: -0.081, 0.472]. However, under wider priors, the evidence in favor of the null hypothesis was more decisive–wide prior: *BF*_*10*_ = 0.39, *δ* = 0.231 [95% CI: -0.065, 0.525] (weak evidence), ultrawide prior: *BF*_*10*_ = 0.30, *δ* = 0.235 [95% CI: -0.054, 0.539] (moderate evidence).

### The influence of the interplay between distance and touch availability on RHI strength: Linear quantile mixed models analysis

An LQMM model for three quartiles of RHI strength— 25^th^ percentile: illusion logit = 0 (score = 0); 50^th^ percentile: illusion logit = 1.25 (score = 1.67); 75^th^ percentile: illusion logit = 2.08 (score = 2.33)—conditional on distance separating hands (D1: close; D2: far), touch availability during illusion elicitation (T1: none; T2: simple; T3: complex), and their interaction (model 1), was fitted on individuals with a random intercept:

(1)illusionLogit∼distance*complexity+∼1|ID

We observed main effects of distance for the 25^th^ and 50^th^ percentiles, which means that, when no touch was applied during illusion elicitation (T1), RHI was significantly attenuated at the far distance (D2) relative to the near distance (D1), but only among weak and moderate responders. For the 75^th^ percentile (strong responders), the effect of distance balanced on the edge of statistical significance. The magnitudes of expected decrease (on the seven-point scale) for the groups were as follows: -2.57 [95% CI: -2.93, -0.95], *p* = 0.009 (for the weak responders), -2.30 [95% CI: -2.85, -0.53], *p* = 0.017 (for the moderate responders), and -1.86 [95% CI: -2.72, 0.17], *p* = 0.069 (for the strong responders). Similarly, the LQMM revealed main effects of touch complexity, but only for the two lower distribution ranges (among the weak and moderate responders) where the illusion was significantly stronger when elicited by tactile stimulation. The gradients of expected increase in RHI vividness at the shorter distance (D1)—as compared to the baseline no touch condition (T1)—were similar for simple (T2) and complex (T3) touch conditions. For simple touch, they amounted to 2.34, [95% CI: 0.66, 2.86], *p* = 0.013 (weak responders, T2), 1.86 [95% CI: 0.30, 2.62], *p* = 0.023 (moderate responders, T2), and 1.54 [95% CI: -0.61, 2.61], *p* = 0.15 (strong responders, T2), whereas for complex tactile stimulation they equaled 2.52 [95% CI: 1.12, 2.90], *p* = 0.004 (weak responders, T3), 2.08 [95% CI: 0.43, 2.75], *p* = 0.019 (moderate responders, T3), and 1.85 [95% CI: -0.92, 2.83], *p* = 0.174 (strong responders, T3).

**Fig 3:**
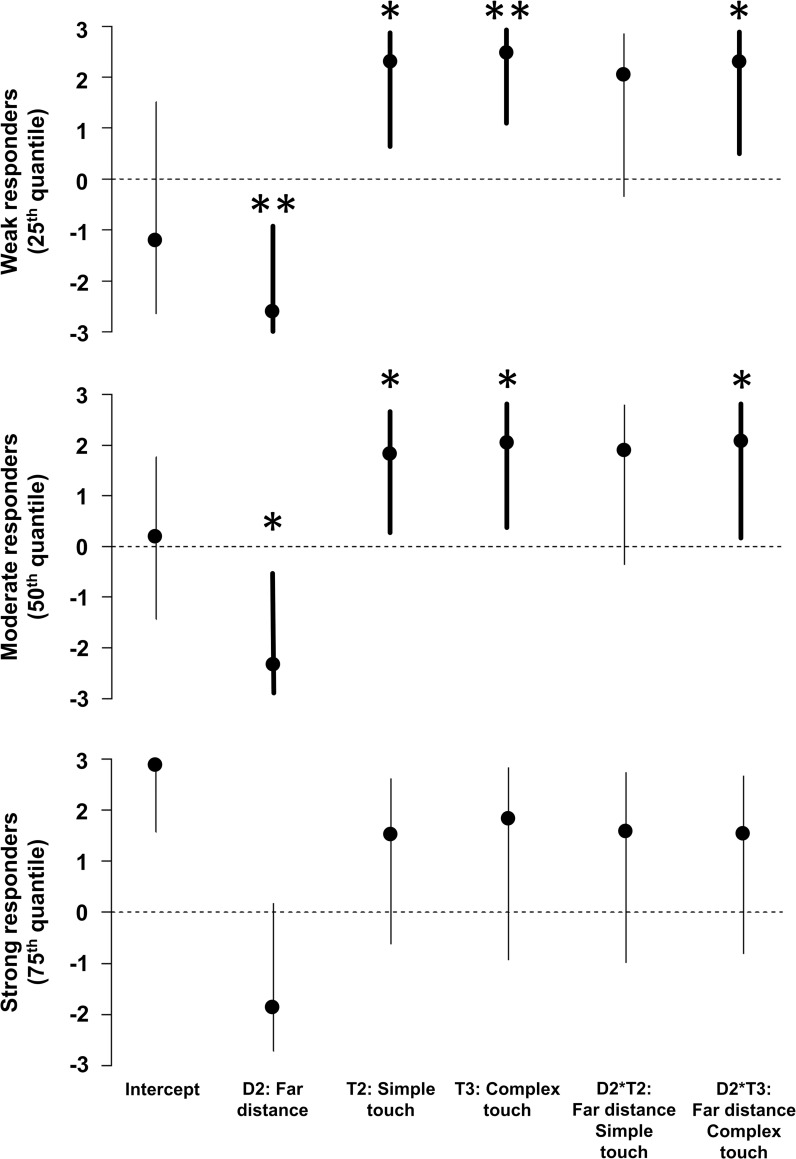
Effects on subjective RHI strength of distance, touch availability/complexity, and their interaction. Effects were estimated by a linear quantile mixed model (LQMM) at three tested percentiles (25^th^–weak responders; 50^th^–moderate responders, 75^th^–strong responders). For main effects, coefficients represent expected change in the outcome variable (RHI strength) for each unit of change in the predictor when the interacting variable is at the baseline level (e.g., expected change with increased distance (D2) when no touch (T1) was applied during illusion elicitation). For interactions, the coefficients reflect the difference in the change of RHI strength with distance (D2), compared to the change when the interacting variable is at the baseline level (T1) [44]. For example, at the 25^th^ percentile, attenuation of the RHI with distance in the complex touch condition (T3) is 0.22, compared to 2.57 at the baseline level (T1)—a decrease of 2.35 (as shown by the D2:T3 coefficient). Taken together, these results show that complex tactile information counteracted the attenuation of RHI strength caused by increased distance only among weak and moderate responders. The simple touch condition showed similar yet non-significant tendencies, which suggests that simplified tactile stimulation may be less potent in counteracting the attenuating effect of increased distance. Bold lines show significant effects. ** *p* < 0.01, * *p* < 0.05. (Note that the unequal confidence interval bounds are due to the non-linear back-transformation to the original scale).

Importantly, in line with our first hypothesis, a significant *far distance–complex touch* interaction was observed (Fig 3), which means that the decrease of RHI strength at the farther distance was lower when the illusion was elicited by complex tactile stimulation. Again, this effect surfaced only among weak and moderate responders, whereas it was not significant in the case of strong responders. The attenuation caused by increased distance—as compared to the T1 condition—was diminished, respectively, by 2.35 [95% CI: 0.51, 2.88], *p* = 0.019 (weak responders), 2.12 [95% CI: 0.19, 2.80], *p* = 0.034 (moderate responders), and 1.55 [95% CI: -0.80, 2.67], *p* = 0.184 (strong responders). The *far distance–complex touch* interaction showed an analogous pattern, but did not reach statistical significance in either of the tested percentiles: 2.08 [95% CI: -0.34, 2.85], *p* = 0.083 (weak responders), 1.93 [95% CI: -0.35, 2.79], *p* = 0.09 (moderate responders), and 1.60 [95% CI: -0.98, 2.73], *p* = 0.211 (strong responders).

### The influence of proprioceptive accuracy/precision on subjective illusion strength in various RHI elicitation conditions

The mean proprioceptive detection threshold was 1.20° (*SD* = 0.5°) with a mean slope of 0.51° (*SD* = 0.06°). Individual slope values and discrimination thresholds were not significantly correlated: *r*_*s*_(38) = 0.22, *p* = 0.164, which suggests that the variability of one’s responses was not associated with one’s accuracy on the hand position discrimination task. Thus, to verify our second hypothesis, we decided to fit two separate LQMM models for the same three quartiles of RHI strength (weak, moderate, and strong responders), each with one additional continuous (centred) predictor—either individual JND threshold (proprioceptive accuracy; model 2) or the slope of the distribution, here encoded as the *sigma* parameter (proprioceptive precision; model 3):

(2)illusionLogit∼distance*complexity*threshold(centred)+∼1|ID

(3)illusionLogit∼distance*complexity*sigma(centred)+∼1|ID

Contrary to our predictions, neither of the models yielded any significant main effects of the newly introduced predictors (threshold: all *p*s > 0.09; sigma: all *p*s > 0.84) or significant interactions between the JND threshold or slope of the distribution of responses and the distance/complexity variables (all *p*s > 0.37) for any of the tested percentiles. In line with model 1, the *far distance–complex touch* interaction was significant among the weak responders—model 2: 2.68 [95% CI: 1.02, 2.96], *p* = 0.009; model 3: 2.66 [95% CI: 1.02, 2.96], *p* = 0.009)—and the *far distance–simple touch* interaction was only on the verge of statistical significance—model 2: 2.33 [95% CI: -0.21, 2.92], *p* = 0.066; model 3: 2.42 [95% CI: -0.26, 2.94], *p* = 0.069. In parallel with model 1, a significant *far distance–complex touch* interaction surfaced also among moderate responders in model 3: 2.45 [95% CI: 0.49, 2.92], *p* = 0.022. These findings indicate the robustness of the interaction effect—particularly among weak responders—showing that the decrease of RHI strength with distance may be prevented with the use of complex tactile stimulation during illusion elicitation.

Finally, in line with the LQMM analyses, additional correlational analyses showed no significant associations between either JND threshold or the variability of responses on the proprioceptive task and RHI strength for any of the six elicitation conditions (for JND threshold, all Spearman's Rhos were: 0.08 < *r*_*s*_ < 0.26, all *p*s > 0.09; and for sigma they were: -0.01 < *r*_*s*_ < 0.27, all *p*s > 0.08; S3 Fig).

### The relation between proprioceptive drift, RHI strength, and proprioceptive accuracy/precision

We found proprioceptive drifts ranging from 0.98 cm to 2.19 cm (see S1 Table for descriptive statistics) depending on the illusion elicitation condition. Our third hypothesis was not supported by the evidence: we did not observe significant correlations between the drifts and subjective measures of the illusion in either of the conditions (-0.04 < *r*_*s*_  < 0.26; *p*s > 0.09; S4 Fig). Similarly, we did not detect any significant associations between the drifts in particular conditions and proprioceptive acuity measures for either proprioceptive accuracy (-0.12 < *r*_*s*_  <  0.24; *p*s > 0.14) or precision (-0.21 < *r*_*s*_  < 0.22; *p*s > 0.17). Surprisingly, proprioceptive drifts in various conditions did not correlate with themselves (all *p*s > 0.13), except for T2 and T3 drifts in the D1 condition: *r*(41) = .36, *p* = 0.016.

## Discussion

In the present study, we examined how sense of body ownership is determined by the spatial properties of visual, tactile, and proprioceptive signals. For this purpose, the RHI was elicited in different conditions varying with respect to the distance separating the hands (i.e., the extent of visuo-proprioceptive spatial divergence) and the informativeness of tactile stimulation (i.e., the presence and complexity of tactile spatial information). We aimed to verify three hypotheses derived from the extended Bayesian model of the RHI [5]. First, we expected that the RHI will be less vivid with increasing distance between hands (i.e., increasing divergence of visual and proprioceptive estimates), but only when tactile information is simplified or altogether absent during illusion elicitation. In line with this hypothesis, we found that the RHI was attenuated with distance only when the illusion was induced either through passive observation of the hand or repetitive (simplified) tactile stimulation. When complex tactile stimulation was applied, the RHI was comparably vivid at both distances, which is in line with our previous findings [8]. The LQMM analyses revealed the diminishing of RHI in the no touch condition to be significantly greater than in the complex touch condition and only marginally greater than in the simple touch condition. This pattern of tactile-dependent reduction of distance effects surfaced in participants generally less prone to the RHI (among the weak and moderate responders), but was not observed in those more susceptible to the illusion (the strong responders).

These findings suggest that the degree of visuo-proprioceptive convergence is a relevant factor for RHI, but its relative impact diminishes in the presence of highly informative tactile stimulation. In our interpretation, increasingly complex visuo-tactile stimulation rigidifies the high likelihood of a common cause of visual and somatosensory signals (leading to RHI), regardless of the exact scale of the visuo-proprioceptive discrepancy (within coupling prior bounds). However, when tactile information is absent or simplified, the proprioceptive modality becomes a valuable source of information for the causal inference processes which identify the boundaries of one’s body (cf. [7]). This pattern of results was explicitly predicted by the extended Bayesian model of the RHI [5], but seems to be hardly accounted for in other competing models. Traditional theoretical approaches to the RHI [48] state that the visuo-proprioceptive recalibration of tactile coordinates to the rubber hand (the “visual capture” of proprioception) occurs *prior to* visuo-tactile integration, either allowing or preventing this integration in advance. If visuo-proprioceptive recalibration operated in such a bottleneck fashion, complex tactile stimulation could not compensate back for visuo-proprioceptive discrepancy. On the other hand, approaches attributing formation of sense of body ownership to cumulative multisensory integration processes [3] conceive of such processes as arising out of different combinations of available sensory information, without the principal dominance of any particular sense. While no sensory domain has been shown to be *necessary* for body transfer phenomena to occur (cf. [3]), our results suggest that relatively unconstrained tactile signals override proprioceptive ones due to their affiliation to a modality of higher spatial resolution. If the multisensory processes which determine body ownership were simply additive, visuo-proprioceptive divergence should also attenuate the RHI elicited by complex tactile stimulation, which was not observed in the present study (see also [8]).

Additionally, LQMM analyses showed that complex tactile information compensates for visuo-proprioceptive divergence only among individuals ranking lower in the distribution of RHI strength–in the 25^th^ and 50^th^ percentiles, that is, among weak and moderate RHI responders. For strong responders, the presumably robust effects of distance and tactile stimulation complexity (as well as their interaction) decline, which indicates that, for such individuals, even imperfect multisensory convergence triggers a convincing illusion. We speculate that strong responsiveness results from strong priors coupling visual and somatosensory signals (for a discussion see [5]), promoting RHI occurrence as long as multisensory stimulation properties do not exceed the spread of the coupling Gaussian (when the distance transcends 30cm or the rubber hand is placed outside trunk-centred peripersonal space, the ownership significantly decreases [10, 11, 27]. However, in more ‘inferentially conservative’ participants, a higher degree of visuo-proprioceptive and/or visuo-tactile convergence (within the coupling prior spread) is needed to tip the inferential balance in favor of the illusion. Therefore, when there is a large distance between hands, the RHI might be triggered only through (complex) tactile stimulation boosting the likelihood of a common cause of the incoming signals.

Our second hypothesis predicted that illusion strength would decrease with greater precision of one's proprioception, but only under the conditions of absent or simplified tactile stimulation (when proprioceptive signals have increased inferential importance). To assess individual proprioceptive competence, we used an elbow joint angular position detection task with sensory conditions mirroring those used in RHI induction. Contrary to our hypothesis, proprioceptive acuity was not correlated with RHI strength in any of the six elicitation modes. Small positive correlations were insignificant for both indicators, that is, for both the just-noticeable-difference threshold (proprioceptive accuracy) and the variability of one’s responses (proprioceptive precision). These results converge with our previous findings [8] and generalize them to another (passive) form of proprioception and a wider range of RHI elicitation conditions. Thus, an accumulating body of evidence suggests that a drop in RHI strength attributable to increased visuo-proprioceptive discrepancy is not weighted by the precision of one’s proprioceptive signals, even if their relative reliability (informativeness) is higher. This seems to contradict Bayesian accounts of body perception, although an alternative explanation might also be offered: since visual spatial acuity is superior to proprioceptive acuity by an order of magnitude [7], the effect exerted by proprioceptive signals on the result of causal inference may be relatively stable regardless of individual proprioceptive abilities. It is also worth noting that proprioceptive measurement paradigms are based on the questionable assumption that task performance reflects signal properties (precision). The question of the role of the signals' reliability in determining body ownership should perhaps be addressed more decisively through direct manipulation of signal properties, such as the addition of visual noise (cf. [49]), thereby enhancing the relative impact of proprioception. Such methods would also yield causal rather than merely correlational evidence.

Finally, we predicted that the strength of the positive association between proprioceptive drift and subjective RHI ratings will be inversely proportional to the amount of available tactile information. Contrary to our hypothesis, we found no associations between these two measures of RHI strength, regardless of the exact mode of RHI induction. This might seem peculiar given the established position of the proprioceptive drift as a “behavioral proxy of RHI” in the literature [22]. However, such results are not uncommon, as no relation between proprioceptive drift and subjective RHI is frequently reported [17–21] and the prevalence of such findings may be underestimated, given that a number of studies do not give information about the relation of these two measures (e.g., [50, 51]). It is also worth noting that, originally, proprioceptive drift was found to be a correlate of illusion prevalence time during a 30 minute period (in %) rather than its vividness [2].

This inconsistent picture of the relation between subjective ratings of RHI and proprioceptive drift might spring from the fact that the latter does not simply reflect a multisensory estimate of the location of one’s hand, but rather is a heterogeneous phenomenon determined by a multitude of extraperceptual factors. Associations between proprioceptive drift and subjective RHI tend to be found with the use of a particular measurement method: the contralateral matching task (i.e., when one reaches towards a position above an ipsilateral hand with a contralateral hand) [22]. The drift has also been shown to be hand-specific, as its magnitude is greater for one’s non-dominant hand [52]. Moreover, it is highly dependent on the way the instruction is phrased [36] and individual hypnotic suggestibility [53, 54], which suggests the involvement of higher-order cognitive processes. Thus, the methodological choices made on the basis of our hypotheses (i.e., choosing a visual judgment task involving the cognitively-mediated assessment of one’s hand’s position) might have actually promoted lower correlations between the two indices of RHI strength.

However, one could reasonably ask whether our main findings could also have been driven by cognitive mediation, given that subjectively assessed RHI strength has also been shown to increase with one’s suggestibility [53, 55] Moreover, when participants are asked about their expectations regarding the experience on an RHI questionnaire, they reproduce the typically-observed pattern of results for synchronous and asynchronous conditions without actually experiencing the illusion [56]. On this basis, it has been suggested that most RHI-based findings (or even the RHI phenomenon itself) are simply driven by demand characteristics [56]. We believe that, in the case of the presented study, this is unlikely for both methodological and theoretical reasons. First, the participants were actually *not* aware that their hands had been displaced and that the illusion was being elicited in two different locations. Second, our hypothesized interaction pattern was far too specific to be identified by a person without academic training in the body ownership field (unlike, for instance, the expected differences between synchronous and asynchronous stroking). Our methodological precautions notwithstanding, we agree that one should not draw far-reaching conclusions based on self-reports alone. Interestingly, a new methodological avenue for RHI research has just been opened: Chancel and Ehrsson [57] used a psychophysical (two-alternative forced-choice) task to determine which of two simultaneously stimulated rubber hands felt more like the participant’s own hand and found a preference for rubber hands placed just 5 cm closer to the participant’s hand. While this finding converges with the attenuation reported in our study (their tactile stimulation patterns would be considered “simple” in our design), it also shows that the spatial constraints of the RHI may be much tighter than previously thought. We believe that this psychophysical approach can be fruitfully coupled with methodological paradigms masking experimental manipulations (i.e., subliminal hand displacements) [23] to further reduce potential suggestibility effects and increase the sensitivity of methods examining the multisensory determinants of body ownership.

In conclusion, we found that the use of complex tactile stimulation during RHI elicitation counters the attenuating effect of increased distance between hands on the strength of the illusion. Our results indicate that the relative impact of proprioception on body attribution processes depends on the availability and informativeness of tactile signals, and is rendered ineffective by complex-structured spatiotemporal visuo-tactile correlations [5]. These findings augment our understanding of body ownership as being dynamically shaped by various combinations of multisensory inputs [3] by showing that feelings of ownership are primarily determined by informative signals from the most relevant sensory domains, rather than an unconstrained accumulation of all sensory evidence.

## Supporting information

S1 Table Descriptive statistics for RHI questionnaire scores (illusion and control) and proprioceptive drift (in cm) in all elicitation conditions. (DOCX)

S1 Fig Distributions of RHI questionnaire illusion scores in all elicitation conditions. The distributions violated normality assumption in all tested conditions, as shown by Shapiro-Wilk normality tests: D1:T1 –W(46) = 0.88, *p* < 0.001; D1:T2 –W(46) = 0.84, *p* < 0.001; D1:T3 –W(46) = 0.80, *p* < 0.001; D2:T2 –W(46) = 0.89, *p* < 0.001; D1:T1 –W(46) = 0.88, *p* < 0.001; D1:T1 –W(46) = 0.85, *p* < 0.001.
(TIF)

S2 Fig RHI questionnaire results–illusion and control scores–for all combinations of within-subject factors. Planned comparisons (Wilcoxon paired signed-rank tests) revealed significant differences between illusion and control scores in all RHI elicitation conditions. Control scores did not significantly differ across conditions. Error bars represent standard errors. *** p < 0.001, ** p < 0.01, * p < 0.05, Bonferroni-corrected.
(TIF)

S3 Fig Associations between proprioceptive acuity indicators and subjective RHI scores. Correlation matrix presents cross-correlations between JND threshold (proprioceptive accuracy), sigma parameter (proprioceptive precision) and RHI scores in particular elicitation conditions. Spearman’s rank correlations are presented as correlation coefficients. *** p < 0.001, ** p < 0.01, * p < 0.05.
(TIF)

S4 Fig Associations between subjective RHI strength and proprioceptive drift. Scatterplots show no significant linear relationships across conditions: D1:T1 –*r*_*s*_(43) = -0.04, *p* = 0.799; D1:T2 –*r*_*s*_(42) = 0.03, *p* = 0.822; D1:T3 –*r*_*s*_(42) = 0.04, *p* = 0.8; D2:T1 –*r*_*s*_(41) = 0.003, *p* = 0.986; D2:T2 –*r*_*s*_(41) = 0.26, *p* = 0.091; D2:T3 –*r*_*s*_(41) = 0.05, *p* = 0.755.
(TIF)
