# Supplementary material for: Tactile information counteracts the attenuation of rubber hand illusion attributable to increased visuo-proprioceptive divergence
Source: PLoS One. 2020 Dec 30;15(12):e0244594. doi: 10.1371/journal.pone.0244594 (PMC7773248; doi:10.1371/journal.pone.0244594)
Supplement: S1 Table — (DOCX) [file pone.0244594.s001.docx]

**Table S1. Descriptive statistics for RHI questionnaire scores (illusion and control) and proprioceptive drift (in cm) in all elicitation conditions.**

|  | **RHI illusion (mean)** | **RHI illusion (SD)** | **RHI control (mean)** | **RHI control (SD)** | **Drift (mean)** | **Drift (SD)** |
| --- | --- | --- | --- | --- | --- | --- |
| **Close distance - no touch**  **(D1:T1)** | 0.41 | 2.23 | -1.26 | 1.60 | 1.32 | 2.16 |
| **Close distance - simple touch**  **(D1:T2)** | 1.72 | 1.23 | -1.14 | 1.55 | 0.98 | 1.93 |
| **Close distance - complex touch**  **(D1:T3)** | 1.86 | 1.25 | -1.04 | 1.59 | 1.74 | 2.24 |
| **Far distance - no touch**  **(D2:T1)** | -0.57 | 2.19 | -1.51 | 1.49 | 1.23 | 2.55 |
| **Far distance - simple touch**  **(D2:T2)** | 0.89 | 1.81 | -1.42 | 1.49 | 2.19 | 2.15 |
| **Far distance - complex touch**  **(D2:T3)** | 1.46 | 1.61 | -1.28 | 1.38 | 2.13 | 2.37 |
