# Supplementary figures and images for: Tactile information counteracts the attenuation of rubber hand illusion attributable to increased visuo-proprioceptive divergence
Source: PLoS One. 2020 Dec 30;15(12):e0244594. doi: 10.1371/journal.pone.0244594 (PMC7773248; doi:10.1371/journal.pone.0244594)

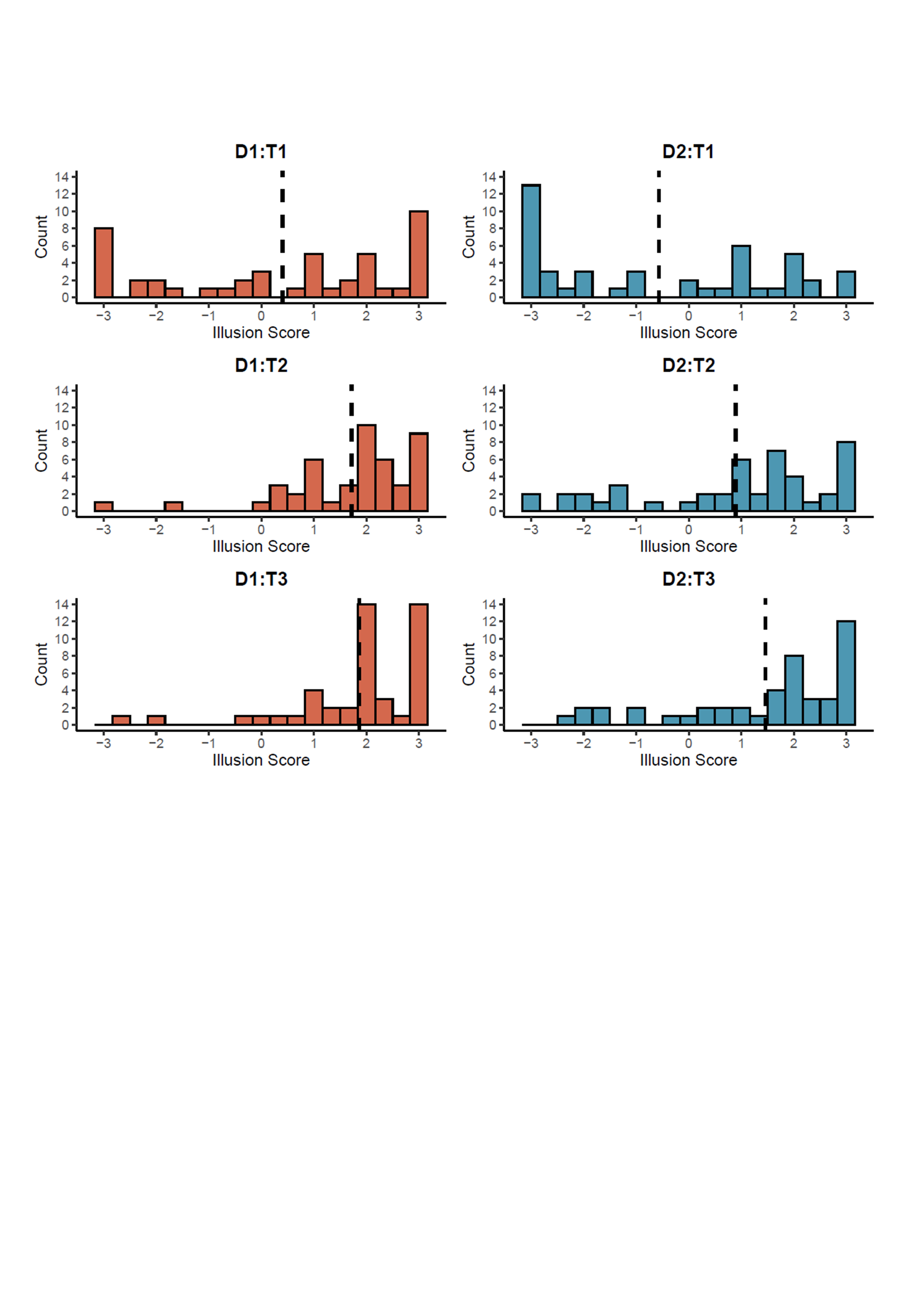

Supplement: S1 Fig — The distributions violated normality assumption in all tested conditions, as shown by Shapiro-Wilk normality tests: D1:T1 –W(46) = 0.88, p < 0.001; D1:T2 –W(46) = 0.84, p < 0.001; D1:T3 –W(46) = 0.80, p < 0.001; D2:T2 –W(46) = 0.89, p < 0.001; D1:T1 –W(46) = 0.88, p < 0.001; D1:T1 –W(46) = 0.85, p < 0.001. (TIF) [file pone.0244594.s002.tif]

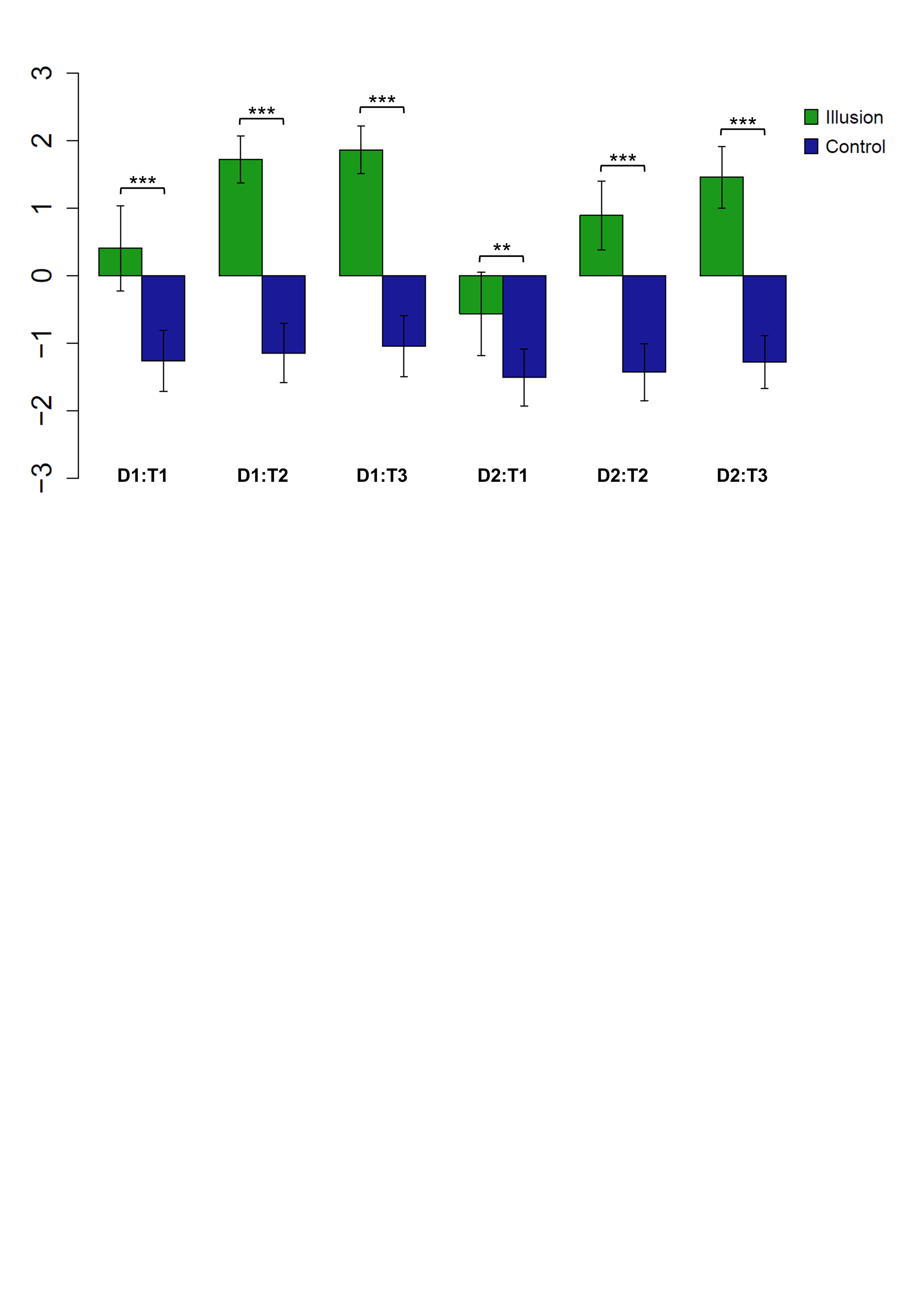

Supplement: S2 Fig — Planned comparisons (Wilcoxon paired signed-rank tests) revealed significant differences between illusion and control scores in all RHI elicitation conditions. Control scores did not significantly differ across conditions. Error bars represent standard errors. *** p < 0.001, ** p < 0.01, * p < 0.05, Bonferroni-corrected. (TIF) [file pone.0244594.s003.tif]

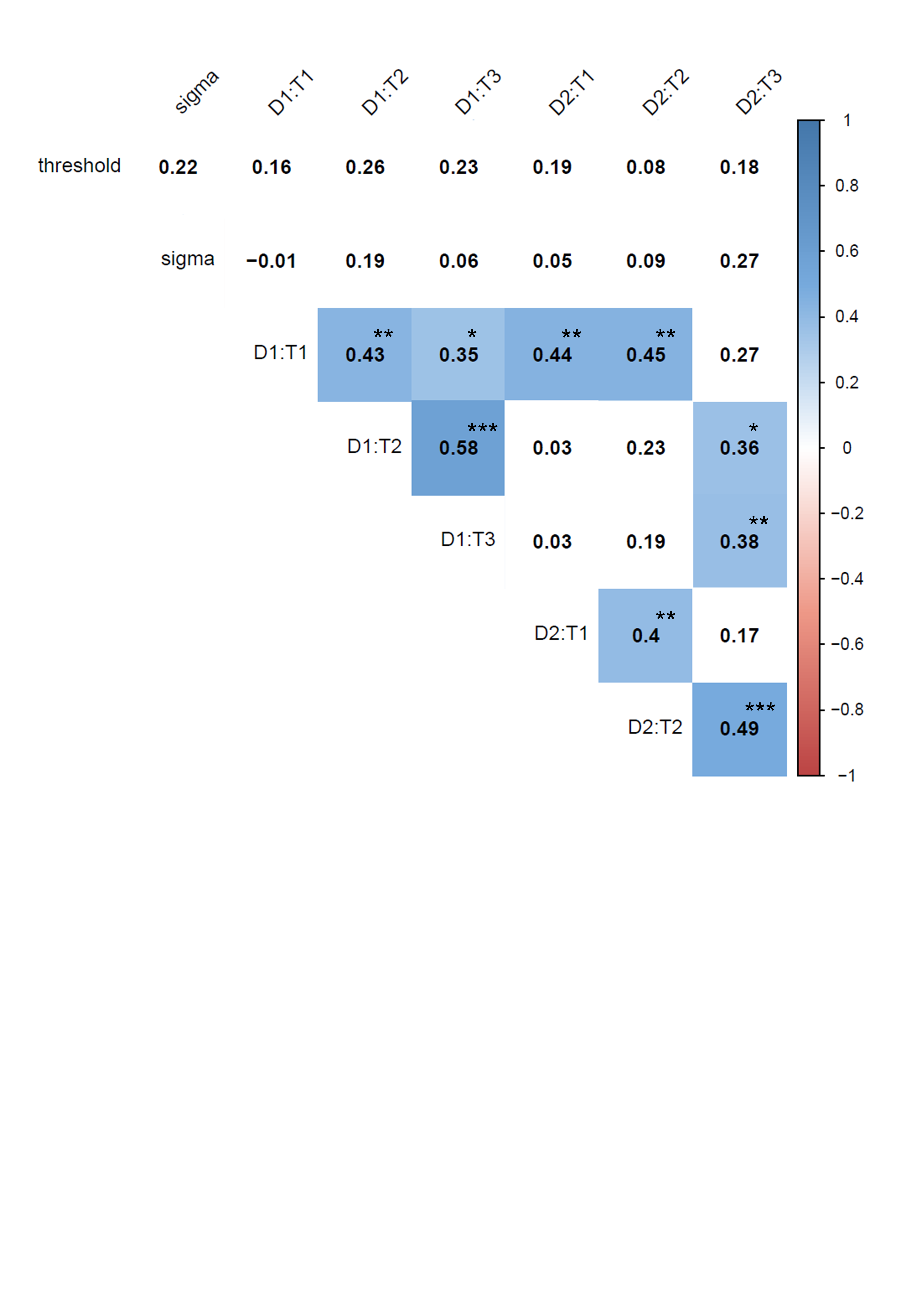

Supplement: S3 Fig — Correlation matrix presents cross-correlations between JND threshold (proprioceptive accuracy), sigma parameter (proprioceptive precision) and RHI scores in particular elicitation conditions. Spearman’s rank correlations are presented as correlation coefficients. *** p < 0.001, ** p < 0.01, * p < 0.05. (TIF) [file pone.0244594.s004.tif]

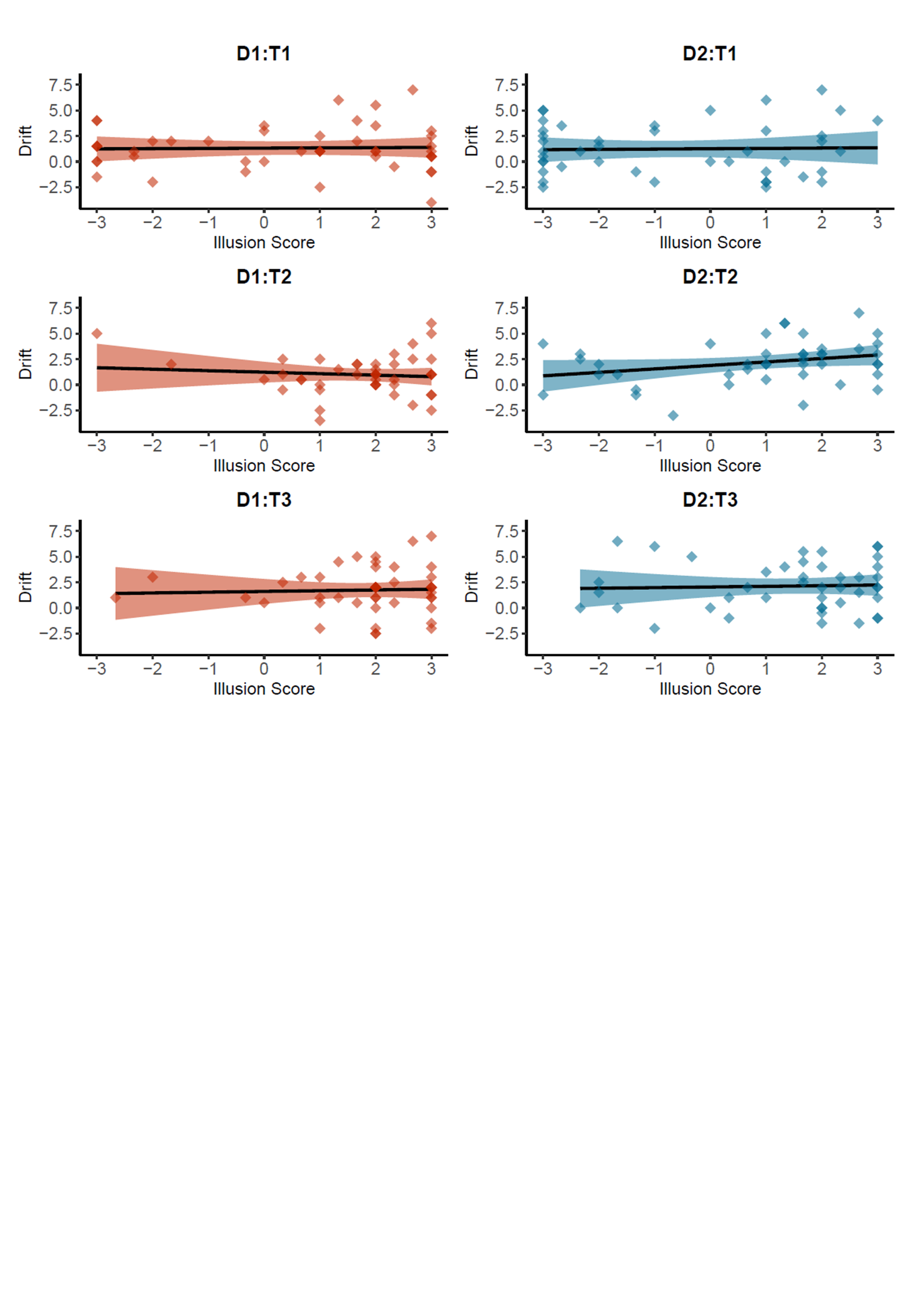

Supplement: S4 Fig — Scatterplots show no significant linear relationships across conditions: D1:T1 –rs(43) = -0.04, p = 0.799; D1:T2 –rs(42) = 0.03, p = 0.822; D1:T3 –rs(42) = 0.04, p = 0.8; D2:T1 –rs(41) = 0.003, p = 0.986; D2:T2 –rs(41) = 0.26, p = 0.091; D2:T3 –rs(41) = 0.05, p = 0.755. (TIF) [file pone.0244594.s005.tif]
